# Electroacupuncture promotes the repair of the damaged spinal cord in mice by mediating neurocan‐perineuronal net

**DOI:** 10.1111/cns.14468

**Published:** 2023-11-10

**Authors:** Rong Hu, Kelin He, Bowen Chen, Yi Chen, Jieqi Zhang, Xingying Wu, Mengting Shi, Lei Wu, Ruijie Ma

**Affiliations:** ^1^ The Third School of Clinical Medicine (School of Rehabilitation Medicine), Key Laboratory of Acupuncture and Neurology of Zhejiang Province Zhejiang Chinese Medical University Zhejiang China; ^2^ Department of Acupuncture and Moxibustion Third Affiliated Hospital of Zhejiang Chinese Medical University Zhejiang China

**Keywords:** electroacupuncture, neurocan, parvalbumin interneuron, perineuronal net, spinal cord injury

## Abstract

**Aims:**

This study aimed to investigate the effect of perineuronal net (PNN) and neurocan (NCAN) on spinal inhibitory parvalbumin interneuron (PV‐IN), and the mechanism of electroacupuncture (EA) in promoting spinal cord injury (SCI) repair through neurocan in PNN.

**Methods:**

A mouse model of SCI was established. Sham‐operated mice or SCI model mice were treated with chondroitin sulfate ABC (ChABC) enzyme or control vehicle for 2 weeks (i.e., sham+veh group, sham+ChABC group, SCI+veh group, and SCI+ChABC group, respectively), and then spinal cord tissues were taken from the T10 lesion epicenter for RNA sequencing (RNA‐seq). MSigDB Hallmark and C5 databases for functional analysis, analysis strategies such as differential expression gene analysis (DEG), Kyoto Encyclopedia of Genes and Genomes (KEGG), gene set enrichment analysis (GSEA), and protein–protein interaction (PPI). According to the results of RNA‐seq analysis, the expression of NCAN was knocked down or overexpressed by virus intervention, or/and EA intervention. Polymerase chain reaction (PCR), immunofluorescence, western blot, electrophysiological, and behavioral tests were performed.

**Results:**

After the successful establishment of SCI model, the motor dysfunction of lower limbs, and the expression of PNN core glycan protein at the epicenter of SCI were reduced. RNA‐seq and PCR showed that PNN core proteoglycans except NCAN showed the same expression trend in normal and injured spinal cord treated with ChABC. KEGG and GSEA showed that PNN is mainly associated with inhibitory GABA neuronal function in injured spinal cord tissue, and PPI showed that NCAN in PNN can be associated with inhibitory neuronal function through parvalbumin (PV). Calcium imaging showed that local parvalbumin interneuron (PV‐IN) activity decreased after PNN destruction, whether due to ChABC treatment or surgical bruising of the spinal cord. Overexpression of neurocan in injured spinal cord can enhance local PV‐IN activity. PCR and western blot suggested that overexpression or knockdown of neurocan could up‐regulate or down‐regulate the expression of GAD. At the same time, the activity of PV‐IN in the primary motor cortex (M1) and the primary sensory cortex of lower (S1HL) extremity changed synchronously. In addition, overexpression of neurocan improved the electrical activity of the lower limb and promoted functional repair of the paralyzed hind limb. EA intervention reversed the down‐regulation of neurocan, enhanced the expression of PNN in the lesioned area, M1 and S1HL.

**Conclusion:**

Neurocan in PNN can regulate the activity of PV‐IN, and EA can promote functional recovery of mice with SCI by upregulating neurocan expression in PNN.

## Graphical abstract

Research mechanism diagram.

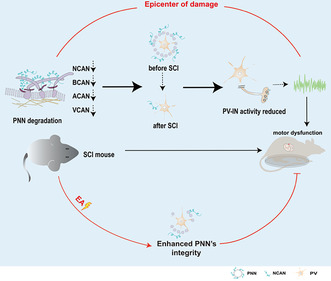

## 1 INTRODUCTION

Spinal cord injury (SCI) is followed by spontaneous anatomical and functional recombination of neural circuits at multiple levels of the nerve axis,^1^ and the environmental factors affecting the damaged nervous system are diverse and complex. Compensatory responses such as neural tissue protection and endogenous repair and arousal after spinal cord injury are limited, which are insufficient to support complete functional recovery. Serious motor and sensory disorders greatly affect the quality of life of patients with SCI.^2^, ^3^ There are about 250,000 to 500,000 cases of SCI in the world every year with an obvious increasing trend year by year. Furthermore, the incidence is predominant in the male population and the average age of patients at the time of injury is 43.7 ± 17.1 years.^4^, ^5^ However, effective strategies to promote functional recovery after SCI are still lacking. Understanding the pathophysiology of SCI and developing new treatment strategies are crucial for patients with SCI.

Perineuronal net (PNN) is the main factors limiting the experience‐dependent remodeling of neuronal circuits^6^ and is a crucial extra‐neural environment in the central nervous system. PNN is a dense reticular structure mainly composed of a hyaluronic acid skeleton rich in chondroitin sulfate proteoglycans, which is widely found around neurons in the central nervous system, especially PV‐IN.^7^ Proteoglycans of PNN include neurocan (NCAN), brevican (BCAN), versican (VCAN), and aggrecan (ACAN).^8^ Physiologically, PNN is involved in maintaining and protecting the activities of neurons, connecting neurons and glial cells, and transmitting signals between them.^9^ Researchers have recently focused on PNN's role in cortical plasticity, neural activity regulation, neural circuits functional reconstruction, and neurodegenerative diseases.^6^, ^10^, ^11^, ^12^ A number of studies have shown that chondroitin sulfate ABC (ChABC), a drug targeting core protein saccharides of PNNs, effectively treats SCI.^13^, ^14^, ^15^ Therefore, modulating PNN is a potentially promising treatment strategy for SCI. However, the specific mechanism of PNN in spinal cord injury remains unclear and needs to be further explored and studied. A recent study identified that the average area under the curve (AUC) of PV+/PNN+ was significantly larger than that of PV+/PNN‐ through in vivo two‐photon modified imaging,^16^ which confirmed the statement that PNN is the key factor to maintain the high‐frequency discharge of Parvalbumin interneuron (PV‐IN).^17^ The relationship between PNN and PV‐IN and their cascade reaction has made significant progress in the studies of brain injury,^18^ optic nerve injury,^19^, ^20^ epilepsy,^21^ and psychiatric disorders.^22^, ^23^ However, the functional repair post‐SCI is still poorly understood.

Electroacupuncture (EA) is a combination of traditional acupuncture and electric current therapy, as an alternative and complementary strategy, has been widely used in the treatment of SCI with multi‐target therapy in clinical practice.^24^, ^25^ Many researchers have proved that EA can not only relieve neuropathological pain after SCI,^26^ promote the repair of motor function of injury,^27^ but also improve intestinal peristalsis^28^ and bladder dysfunction^29^ caused by injury. It plays a particular role in the process of neuronal repair post‐SCI.^30^, ^31^, ^32^ However, the mechanism of EA therapy to improve the repair of nerve function after SCI is complex and diverse, which still needs further study. This study first clarified the regulatory effect of PNN on inhibitory interneurons in SCI tissues by RNA‐seq analysis and suggested that NCAN may be an important factor for the action of PNN through a protein–protein interaction network. Therefore, using NCAN interfering virus intervention combined with electrophysiology and molecular biology to investigate the vital role of NCAN, we found that NCAN in PNN in an SCI model can modulate inhibitory interneurons PV‐IN. EA may promote functional repair after SCI through NCAN in PNN.

## 2 METHOD

### 2.1 Materials and methods

#### 2.1.1 Establishment and data analysis of RNA‐Seq

cDNA library construction, library purification, and transcriptome sequencing were performed according to the instructions of Wuhan Huada Sequencing Company (www.genomics.org.cn). Enriched gene sets with up‐regulated and down‐regulated genes were identified from MSigDB Hallmark and C5 databases for functional analysis. Differentially expressed genes (DEGs) were filtrated with fold change = 0.5 and *q*‐value <0.05 by R package DESeq26. NES >1, *p* < 0.05, and FDR >0.25 as the screening criteria for gene set enrichment analysis (GSEA) were selected in MSigDB for the kegg. c2. v7.4 gene set. Analysis was executed by the clusterprofifiler package in R, and visualization was carried out using the enrichplot package. The basic information of the sample is shown in Table 1.

**TABLE 1:** Effectiveness of RNA‐Seq samples.

Sample	Total raw reads (M)	Total clean reads (M)	Total clean bases (Gb)	Clean reads Q20 (%)	Clean reads Q30 (%)	Clean reads ratio (%)
Sham+Veh1	23.75	23.67	1.18	98.03	93.7	99.67
sham+Veh2	23.75	23.66	1.18	97.85	93.12	99.61
sham+Veh3	23.92	23.82	1.19	96.6	90.79	99.57
SCI+Veh1	23.92	23.81	1.19	96.53	90.55	99.55
SCI+Veh2	21.03	20.93	1.05	96.57	90.72	99.56
SCI+Veh3	18.77	18.69	0.93	96.5	90.5	99.56
sham+ChABC1	23.92	23.82	1.19	96.52	90.5	99.57
sham+ChABC2	23.92	23.82	1.19	96.66	90.89	99.57
sham+ChABC3	23.92	23.82	1.19	96.6	90.73	99.58
sham+ChABC4	21.29	21.2	1.06	96.6	90.77	99.57
sham+ChABC5	23.92	23.82	1.19	96.58	90.68	99.56
SCI+ChABC1	23.92	23.82	1.19	96.63	90.83	99.55
SCI+ChABC2	23.92	23.82	1.19	96.97	91.69	99.55
SCI+ChABC3	23.92	23.82	1.19	96.94	91.62	99.57
SCI+ChABC4	23.92	23.82	1.19	97.02	91.82	99.56
SCI+ChABC5	23.92	23.82	1.19	96.98	91.76	99.55

### 2.2 Animals

Eight‐week‐old mice (C57BL/6J PV‐Cre PV‐Cre:Ai9) were included in this experiment. The PV‐Cre mice (StockNo: 017320, MGI: 3590684) and PV‐cre:Ai9 mice were provided by the Key Laboratory of Acupuncture Neurology Research of Zhejiang Province. C57BL/6J was purchased from Shanghai BK Company by Animal Experimental Center of Zhejiang Chinese Medical University and raised in the center. Among them, adult male PV‐Cre mice were used in calcium imaging experiments for studying activity specifically in PV+ cell in vivo. All adult male PV‐Cre:Ai9 mice (Bred by mixed‐sex PV‐Cre mice (StockNo: 017320) and Ai9 mice (StockNo: 007909)) were used in immunofluorescence for visualization of PV+ cells in vivo. Male C57BL/6J mice have been used for western blotting, behavioral, and other experimental studies. Animals were housed in standard environments (12/12 h light/dark cycle) with five animals per cage and free access to food and water. All animal experiments were approved by the Animal Ethics Committee of Zhejiang Chinese Medicine University (I ACUC‐20220117‐11). All methods were carried out in accordance with relevant guidelines and regulations.

### 2.3 SCI model preparation

The model of spinal cord injury was established by impinging the T10 spinal cord with the Infinite Horizon Impactor (Instrumentation, IH0400). Specifically, mice anesthetized with 0.3% pentobarbital sodium (50 mg/kg) were surgically exposed with a laminectomy to expose the T10 spinal cord and subjected to a 50 kdyn moderate injury with impactor.^33^ The wound was subsequently sterilized and sutured layer by layer. All the mice were reared in cages after awakening on the thermostatic operating table. Mice with Basso Mouse Scale (BMS) motor function score greater than 2 points were considered as modeling failure, excluded and supplemented with the same conditions. Laminectomy was performed only in the sham group. Assisted urination once a day after the operation until the urination reflex recovered.

### 2.4 Stereotaxic injection of ChABC

The mice in the operational group were injected ChABC (50 U/μl; 10 μL; Sigma‐Aldrich, C3667), which was diluted by 0.01% bovine serum albumin, into subarachnoid space in the same day and again every other week. The control group was injected equivalent 0.01% bovine serum albumin slowly.

### 2.5 Virus injection

After the animal was deeply anesthetized with 0.3% pentobarbital sodium (50 mg/kg) 3 weeks prior to modeling, the T9, T10 spinal junction was exposed, and the animal was fixed on a stereoscope. A Hamilton syringe was used to inject the virus solution around the central gray matter ducts on the left and right sides of the spinal cord. The injection dose was 250 nL per side, the speed was 100 nL/min, and the depth was 0.7 mm. The needle is left in place for 5 min to prevent spillover of the virus after the injection is finished.

### 2.6 Fiber optic calcium imaging and analysis

Three weeks before modeling, rAAV‐CAG‐FLEX‐jGCaMP7f‐WPRE‐SV40pa or rAAV‐CAG‐FLEX‐EGFP‐WPRE‐SV40pa, was injected with a Hamilton injector at the T9‐10 junction of the spinal cord, with 250 μL of each, 100 nL/min, and the depth was 0.7 mm. The model was successfully built, and the optical fiber was placed. After the T10 spinal cord was fully exposed, a specially shaped 0.01 mm thick titanium plate with a perforated hole was fixed to the vertebral body and soft tissue using non‐absorbable sutures. Optical fibers with a diameter of 200 μm and a length of 2 mm (with a stereolocator and optical fiber fixer) were then placed under a microscope at the central canal of the spinal gray matter and fixed with glue and dental cement. GCaMP7f is excited at two wavelengths (470 nm, calcium‐related signal, and 405 nm isometric control), reflected to dichroic mirror by amplitude modulation signal and coupled to fiber for calcium imaging fiber photometry recording. Finally, data were imported into MATLAB environment (MathWorks), and off‐line analysis was carried out using self‐developed code. The neuronal activity induced by the harmful stimulation of the paw pinching from the tweezers was recorded, and the neuronal activity was analyzed in the first 2 s and after 10 s of each stimulation.

### 2.7 Electroacupuncture therapy

Electroacupuncture was performed on 24 h after successful modeling. In short, to prevent damage from random movements during the electroacupuncture, the mouse was placed in a special harness to restrict its physical movement, and the T9‐T11 spine was exposed. 0.16 × 7 mm (Zhongguo Taihe; Tianjin, China). Acupuncture was used to insert the “Jiaji” points (EX‐B2, according to atlas of acupuncture and moxibustion points on animals by the Chinese Acupuncture and Moxibustion Society and experimental acupuncture and moxibustion) on both sides of T9 and T11, and the upper and lower ends were connected to the positive and negative poles of the EA therapeutic apparatus (HANS200A; Nanjing, China). 2/100 Hz sparse and dense waves and 0.2 mA current stimulation were carried out for 20 min, once a day for 14 days or 4 weeks. During electroacupuncture treatment, the animals remained calm and awake without obvious stress. The sham electroacupuncture group received the same restraint except that the needle punctured the skin and connected the electroacupuncture apparatus with no electricity.

### 2.8 Western blotting

Whole cell lysate was prepared from a 0.5 cm fragment of injured spinal cord containing the injured site. The protein was isolated and homogenized by release precipitation‐free (RIPA) buffer solution. The proteins were decomposed on 8% sodium dodecyl sulfate‐polyacrylamide gel (SDS‐PAGE) and transferred to polyvinylidene fluoride (PVDF) membranes. Blots were reacted with primary antibodies for NCAN (1:500, Abcam), BCAN (1:500, Abcam), ACAN (1:500, millipore), VCAN (1:500, millipore), GAD (1:1000, Abcam), or beta‐actin (1:5000, Cell Signaling) were incubated overnight at 4°C. After incubation with the corresponding horseradish peroxidase‐conjugated secondary antibody (1:2000, Abcam) for 2 h under RT, the bands were visualized using the Bio‐Rad Image Lab system, and gray value analysis was performed using ImageJ software.

### 2.9 Immunofluorescence

The prepared spinal cord tissue was taken from the refrigerator at −80°C and 20 μm spinal cord sections were prepared using the frozen sectioning mechanism. After the spinal sections were rewarmed in a 37°C thermostatic water bath for 1 h, TBST slides were washed six times, sealed in TBST with 10% goat serum and 0.1% Triton X‐100 for 1 h, and then incubated with primary antibody at 4°C overnight. The primary antibodies used for staining were rabbit anti‐cFos (1:500, Abcam), Wisteria Floribunda Lectin (WFA, 1:100, Thermo Scientific). The next day, after rewarming for 1 h and washing TBST six times, the corresponding secondary antibody (Alexa Fluor™ 647, 1:500, Jackson; or 1:100, Thermo Scientific) was incubated in a 37°C constant temperature bath for 2 h. The excess secondary antibody was washed away, and the tablet was sealed. Finally, the scanning machine was used for visualization, and the positive cells were quantified by Photoshop.

### 2.10 Quantitative real‐time polymerase chain reaction (qRT‐PCR)

Total RNA was extracted from a 0.5 cm fragment of injured spinal cord (or normal spinal cord) using TRizol. After reverse transcription according to the reverse transcriptase kit (TaKaRa) procedure, real‐time PCR was performed for the target gene using SYBR Green (Monad) on the Roche PCR system (PCR480). Gene expression was calculated using 2^−ΔΔCT^ method, and the final data were normalized by beta Actin. Primer sequence is shown in Table 2.

**TABLE 2:** Sequences of the primers used for qRT‐PCR.

Primers	Forward	Reverse	Amplicon size (bp)
ACAN	TGAGGACCTGGTAGTGCGAGTG	GCCTGGGCGATAGTGGAATACAAC	100
NCAN	GGAGTAGATATGGAAGCGACAA	TGTCGACCATTCAACCCTTTAA	95
VCAN	GGATGGTGTTGTGTTTCACTAC	CATCACACTGCTCAAATCCATC	152
BCAN	GCACCACCTACCGAGTCCTCAG	GGTCCTCCTGCTCCTTCTCTTCC	103
Gad1	CAATTCAGTCACCTGGAACCCTCAC	CATACTGCTTGTCTGGCTGGAAGAG	140
Gad2	CAGTGTGGACGCCATGTGGATG	AATGTGTGCCTCAAACCCAGTAGTC	81
Gabrg	ATTCGTCCCAGATCAGCAACCATTC	TCCTTGCCATCCAAACACTCATAGC	95
Gabrd	TTGCCCACTTCAATGCCGACTAC	GCGTTCCTCACATCCATCTCTGC	88
Gabrb2	GTGCCTGACACCTACTTCCTGAATG	GCAGCCGTAGTTGTGATTCTGAGG	128
Gabra1	ACTGCTGGACGGTTATGACAATCG	GGTCTGAAACTGGTCCGAAACTGG	104
Gabra3	AGTCCTGCTGAGACCAAGACCTAC	ATGGCAAAGAGCACAGGGAAGATG	83
Pvalb	GAGGTGAAGAAGGTGTTCCATA	TCTTTAGCAGACAAGTCTCTGG	119

### 2.11 Behavioral test

#### 2.11.1 Basso mouse scale motor scale

Basso mouse scale (BMS) was used to evaluate the recovery of hind limb motor function. A score of 0 on this scale indicates complete paralysis of the hind limb, and a score of 9 indicates complete normal movement of the hind limb. Among them, 0–2 points were used to evaluate the ankle joint movement, 3–4 points were used to evaluate the mice's hind limb support and forward stepping, and 5–8 points were used to evaluate the position of the hind paw, the coordination of the front and rear limbs and the stability of the trunk during the stepping process. Each animal was scored independently before surgery and at different time points after injury in a blind method by an uninformed and familiar observer.

### 2.12 Gait analysis

The DigiGait gait analysis system is the only one that uses real‐time ventral imaging technology and is widely used to study motor function in models of nervous system injury, muscle‐related injury, and trauma. A high‐speed digital video camera continuously photographed the ventral side of the animal while the mice walked on a transparent walkband at a speed of 10 cm/s, generating “digital paw prints” and dynamic gait signals. The gait signals of each paw included the contact duration between the sole of the foot and the walking belt in the standing state and the separation duration between the sole and the walking belt in the swaying state. Each mouse underwent adaptive running on a conveyor belt before undergoing surgical treatment or intervention. Finally, the corresponding results generated by the system were analyzed.

### 2.13 Electromyographic (EMG)

Nicolet (Nicolet EDX:Viking QUEST; US) collected EMG and spinal evoked potential from the gastrocnemius muscle of the left lower limb in mice. Mice (*n* = 6 in each group) were fitted with a special condom, their limbs were removed and fixed, and the hair on the spinal cord of T8‐T10 and the gastrocnemius muscle of the left lower limb were shaved. Simply put, the recording electrode was inserted into the gastrocnemius muscle of the left lower limb, and the unit myoelectricity within the range of 20–500 Hz was collected for 15–20 consecutive times, and the average value was taken. When stimulating electrode was inserted into the spinal nerve at T8‐T9, and the recording electrode was inserted into the gastrocnemius muscle of the left lower limb, the spinal evoked potential was detected by the VikingSelect experimental system. The latency time and amplitude were recorded. Single stimulus parameter (duration 0.1 ms, frequency 1 Hz, current 12.5 mA).

### 2.14 Evaluation of mechanical and thermal hyperalgesia

At the 4th week after operation, mechanical pain in the left hind leg was measured. Before the experiment, mice were placed in a separate plexiglas–glass cage (size: 20 × 10 × 10 cm) with a wire mesh (mesh size: 0.5 × 0.5 cm) at the bottom and adapted to the environment for 15 min. The 50% paw withdrawal mechanical threshold (50% PWTs) was assessed using the up and down method. Place each von Frey hair on the sole surface of the left hind paw for 2–5 s with at least 1 min between stimuli. Positive responses to von Frey's hair stimulation include rapid withdrawal, licking and/or paw shaking. All animals were tested for fitness three times before surgery.

Thermal withdrawal latency (TWL) was obtained by using the hot/cold plate (35,100, Ugo Basile, Italy). The hot plate was set at a constant temperature of 50°C, and a cutoff time of 30 s was set to avoid tissue damage. Rapid retraction or shaking of the hind paw was defined as a positive response, and the latency time of the response was recorded as a thermal threshold for pain. 3 days before surgery, all animals were acclimated for 3 min a day.

### 2.15 Statistical data

All analyses and graphs were captured using GraphPad software or MATLAB. We used the Shapiro–Wilk test to assess the normality of the distribution of continuous variables, and Student's *t*‐test (two‐tailed) and one‐way analysis of variance, followed by Tukey's post hoc test, were used to compare the means of two and multiple groups, respectively, for normally distributed data. Alternatively, the Mann–Whitney *U*‐test and the Kruskal–Wallis H test were used to compare two or more sets of data with non‐normal distributions, respectively. All results are expressed as mean ± SEM, *p* < 0.05 was considered statistically significant.

## 3 RESULTS

### 3.1 The expression of PNN decreased in the epicenter of injured spinal cord

**FIGURE 1:**
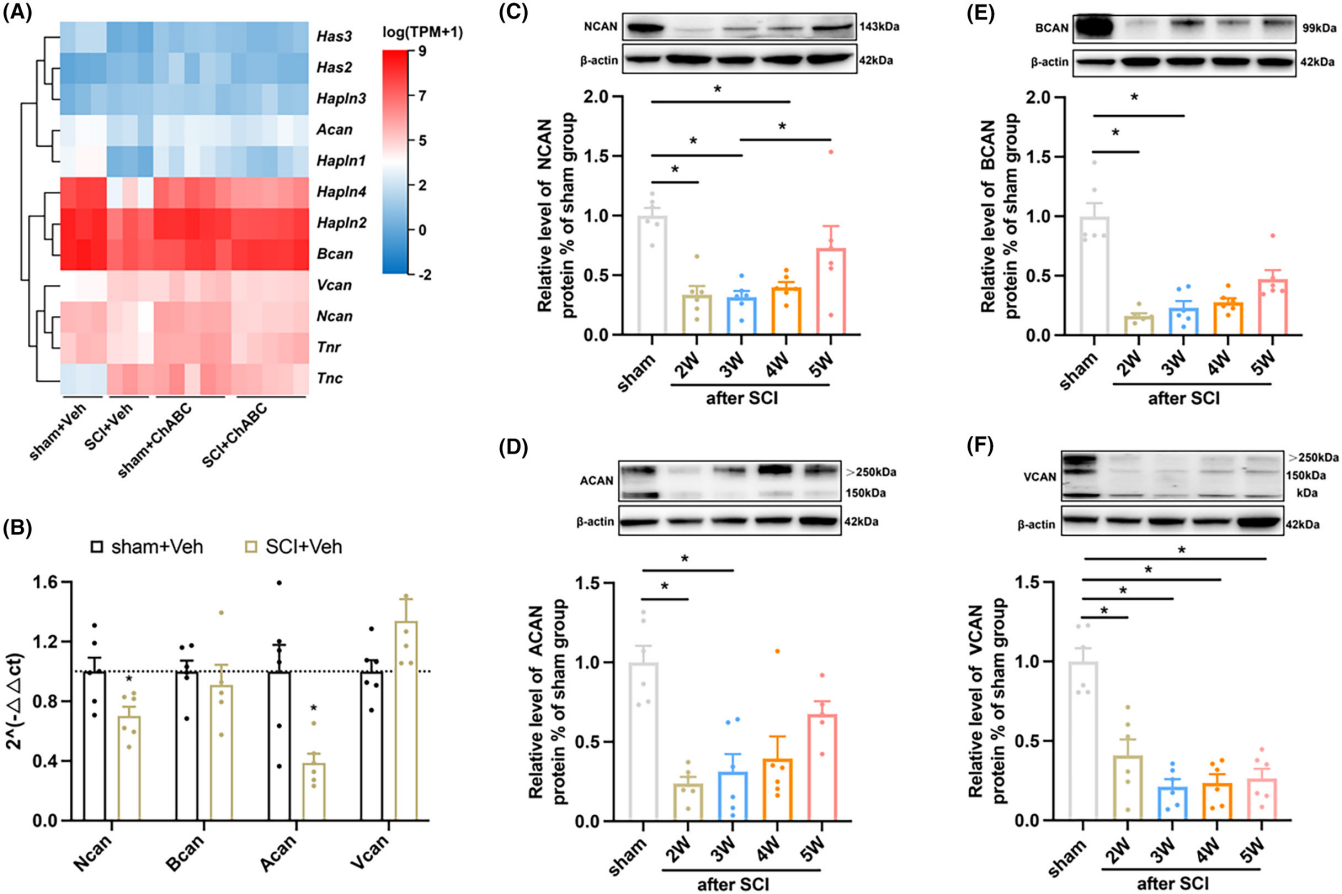
PNN is degraded after SCI. (A) The gene clustering heat map of RNA‐Seq indicated the expression of PNN structural protein in T10 spinal cord after 14 days of intervention; (B) PCR quantization of PNN core proteoglycan gene expression in the epicenter of spinal cord injury on Day 14 (*n* = 6 per group); (C–F) Blot represents banding and quantification of PNN core proteoglycans in spinal cord tissue at different time points after spinal cord injury: The results showed that the protein expression of PNN core proteoglycan in injury epicenter was significantly reduced (*n* = 6 for each time point). Values are presented as the Mean ± SEM. * indicates *p* < 0.05 vs. the sham group. BCAN: brevican; ACAN, aggrecan; ChABC: Chondroitinase ABC; NCAN: neurocan; VCAN: versican.

The mice with a successful SCI model developed motor dysfunction in lower limbs (Figure S1). We randomly divided mice into sham+veh group, sham+ChABC group, SCI+veh group, and SCI+ChABC group for RNA‐Seq detection. Performing cluster analysis (Figure 1A) revealed that the expression of the other major structural protein genes of PNN was significantly reduced at the epicenter of SCI except *Vcan* and *Tnc*. In order to verify the accuracy of RNA‐Seq, we performed PCR detection on several core protein‐coding genes of PNN, and the results were consistent with RNA‐Seq (Figure 1B). To determine whether PNN at the epicenter of injury participated in the pathological mechanism of SCI, western blot was applied, and the results showed that the protein levels of core proteoglycans (NCAN, BCAN, ACAN, and VCAN) of PNN were significantly reduced (Figure 1C–F).

### 3.2 PNN is involved in the functional regulation of GABAergic neurons

**FIGURE 2:**
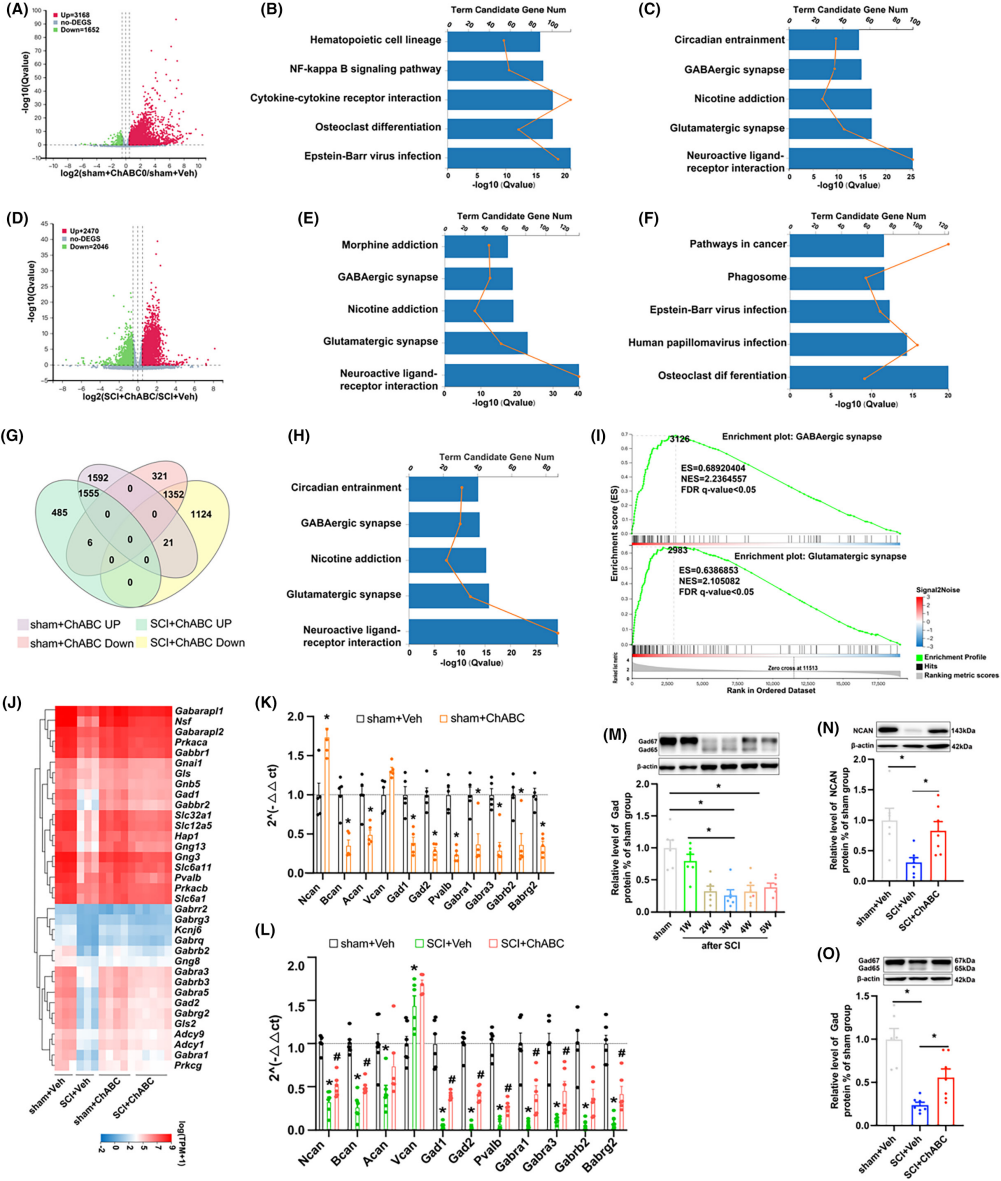
Role of PNN in spinal cord is closely related to the function of GABAergic neurons. (A, D) Differential gene heat maps of sham+Veh/sham+ChABC and SCI+Veh/SCI+ChABC: The results showed that 3168 genes were up‐regulated, and 1652 genes were down‐regulated in the sham+ChABC group compared with the sham+veh group (A), compared with the SCI+veh group, 2470 genes were up‐regulated, and 2046 genes were down‐regulated in the SCI+ChABC group (D); (B, E) shows the first five pathways in which the ascending differential gene KEGG was significant; (C, F) The first 5 pathways in which KEGG was significantly enriched in sham+Veh/sham+ChABC and SCI+Veh/SCI+ChABC; (G) Intersection of sham+Veh/sham+ChABC and SCI+Veh/SCI+ChABC differential genes; (H) The first 5 pathways of KEGG enrichment in 1325 genes intersected by the decreased differential genes in sham+Veh/sham+ChABC and the increased differential genes in SCI+Veh/SCI+ChABC; (I) The first two significant GSEA of 1325 intersecting genes; (J) Clustering heat maps of GABAergic synapse related genes in sham+Veh sham+ChABC SCI+Veh and SCI+ChABC groups by RNA‐Seq; (K) PCR quantification of PNN core proteoglycan and some GABAergic neuron‐related genes in sham+Veh and sham+ChABC groups at 14‐day injury epicenter (*n* = 5 per group); (L) PCR quantification of PNN core proteoglycan and some GABAergic neurons‐related genes in sham+Veh, SCI+Veh and SCI+ChABC groups at 14‐day injury epicenter (*n* = 6 per group), values are presented as the Mean ± SEM. * indicates *p* < 0.05 vs. the sham+Veh group, #*p* < 0.05 vs. the SCI+Veh group; M: The blotting of GAD at different time points after SCI represented banding and quantification(*n* = 6 per group); (N, O) Representative blot bands and quantification of NCAN and GAD at 14‐day injury epicenter in sham+Veh, SCI+Veh, and SCI+ChABC groups (*n* = 6 for each time point). Values are presented as the Mean ± SEM. * indicates *p* < 0.05. ACAN, aggrecan; BCAN: brevican; ChABC: Chondroitinase ABC; GAD: glutamic acid decarboxylase; NCAN: neurocan; VCAN: versican.

Differential gene analysis was performed for sham+veh group, sham+ChABC group, SCI+veh group, and SCI+ChABC group. We found that 3168 genes were up‐regulated and 1652 genes were down‐regulated in the sham+ChABC group compared with the sham+veh group (Figure 2A). Compared with the SCI+veh group, 2470 genes were up‐regulated and 2046 genes were down‐regulated in the SCI+ChABC group (Figure 2D). The most significant accumulation pathways of upper and lower regulation genes were analyzed using the Kyoto Encyclopedia of Genes and Genomes (KEGG) (Figure 2B,C,E,F). KEGG analysis was performed on the standard 1325 song gene of down‐regulated genes in the sham+ChABC group and up‐regulated genes in SCI+ChABC. The five most significantly enriched pathways were neuroactive ligand–receptor interaction, glutamatergic synapse, nicotine addiction, GABAergic synapse, and circadian entrainment (Figure 2G,H). Meanwhile, gene set enrichment analysis (GSEA) suggests that GABAergic synapse plays a central role (Figure 2I). Then cluster analysis and PCR verification were performed on GABAergic synapse‐related genes (Figure 2J–O). It was found that GABAergic synapse‐related gene expression generally decreased after PNN was destroyed due to SCI or ChABC enzyme degradation of normal spinal cord. Blotting also showed down‐regulation of the inhibitory gamma‐aminobutyric acid synthetase GAD after SCI. Amazingly, ChABC intervention reversed these declines in the injured spinal cord.

### 3.3 NCAN may be an important regulator of PNN in the spinal cord

The PPI interaction network between major structural protein genes of PNN and GABAergic synapse pathway enrichment genes was applied (Figure 1K), and it was found that Acan Bcan Ncan in PNN was correlated with GABAergic synapse through pvalb (Figure S2). Cluster analysis of RNA‐Seq and PCR showed that Ncan gene expression was up‐regulated in the normal spinal cord subjected to ChABC enzymatic intervention, decreased in the injured spinal cord, and reversed the downward trend in the injured spinal cord treated with ChABC enzyme (Figures 1A and 2K,L). However, the expression trend of other major structural protein genes of PNN was consistent between ChABC‐treated injured spinal cord and normal spinal cord, while Ncan showed an opposite trend (Figure 2K,L).

### 3.4 PNN degradation decreased the activity of PV‐IN

**FIGURE 3:**
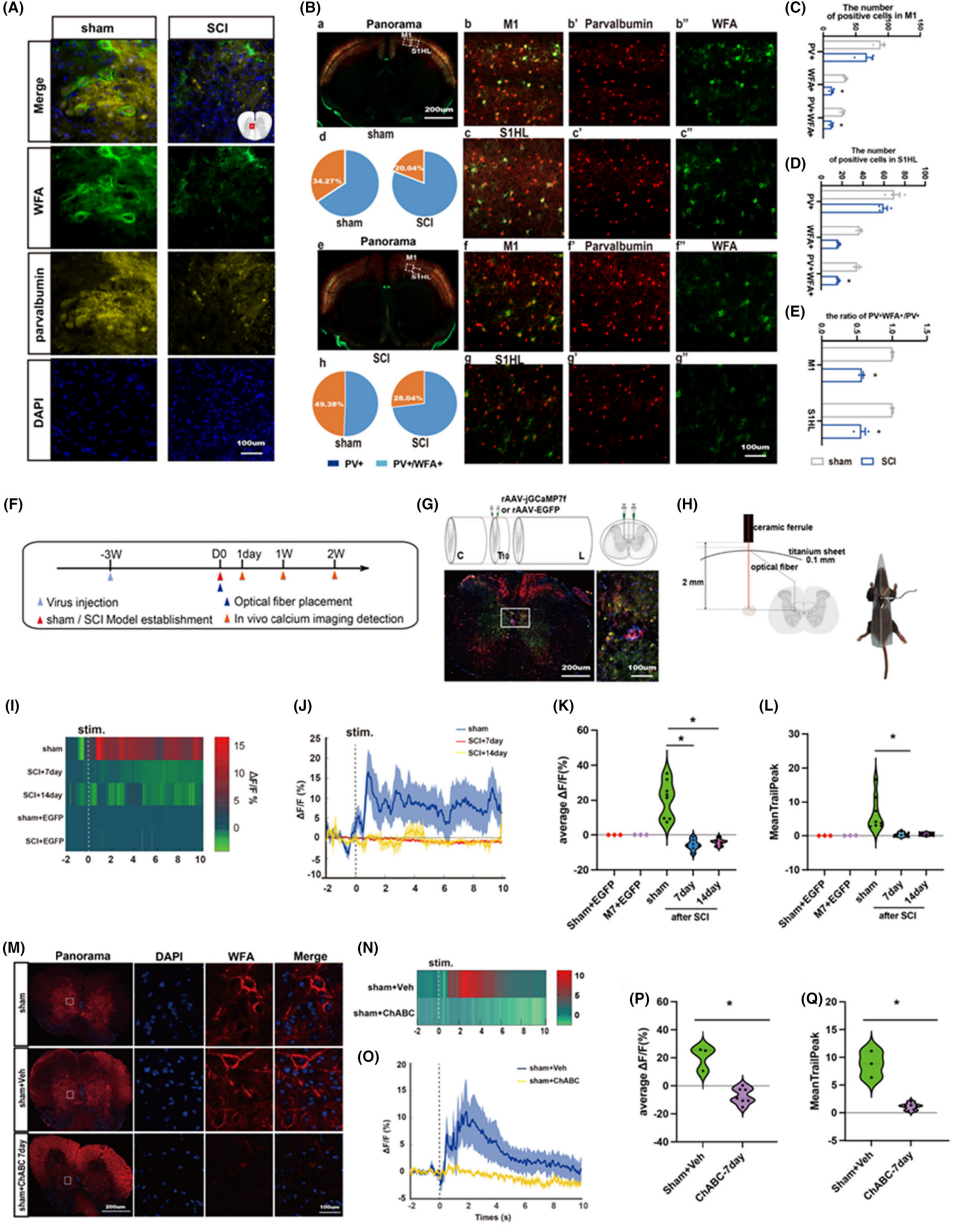
Destruction of PNN results in the decrease of PV‐IN activity. (A) Immunofluorescence expression of WFA (green) and parvalbumin (yellow) in central tubule of spinal gray matter in sham group and SCI group on Day 14. Nucleus (blue); (B–E) Immunofluorescence expression and quantification of WFA (green) and parvalbumin (red) in the M1 and S1HL of the cerebral cortex in sham group and SCI group at Day 14 (*n* = 3 per group). Values are presented as the Mean ± SEM. * indicates *p* < 0.05 vs. the sham group, (F) Experimental timeline; (G, H) A schematic of calcium imaging virus injection with specific PV‐IN labeling, and a fluorescence representation of the virus transfection effect; (I, J) Recorded of the activity of PV‐IN in the spinal cord of mice in each group during stimulation of the hind foot; (K, L) Statistical graph of average △F/F and meantrailpeak of calcium imaging at the epicenter of spinal cord injury on both Day 7 and Day 14; M: Immunofluorescence expression map of PNN (red) in spinal cord tissue at Day 7 after treatment with ChABC or control solvent. Nucleus (blue); (N, O) Representative diagram of PV neuron activity in the spinal cord of mice after stimulation of the hind foot on the 7th day after intervention with ChABC or control solvent; (P, Q) Statistical plots of mean △F/F and meantrailpeak of calcium imaging at spinal cord injury center on Day 7 after ChABC or control solvents intervention: The results showed that PV neuron activity decreased significantly after ChABC intervention (*n* = 6 ~ 8 per group). Values are presented as the Mean ± SEM. * indicates *p* < 0.05. ChABC: Chondroitinase ABC; WFA: wisteria floribunda agglutinin.

By immunofluorescence, we found that compared with the sham group, the expression of wisteria floribunda agglutinin (WFA), a PNNs‐specific marker and the important inhibitory parvalbumin GABA interneurons were down‐regulated, and its co‐expression was significantly reduced in the central tubule of the spinal cord at the epicenter of the injury (Figure 3A). At the same time, we also observed the changes in WFA and parvalbumin in the primary motor cortex (M1) and the primary sensory cortex of the hind limb (S1HL). The results showed that the changing trend of the cerebral cortex was consistent with that of the injured spinal cord. Compared with the sham group, the expressions of WFA and parvalbumin in the model at Week 2 were down‐regulated, and their co‐expressions were also significantly decreased. In the sham group, WFA+/PV+ accounted for 34.27% in M1 and 49.38% in S1HL. In the model group, WFA+/PV+ decreased to 20.04% in M1 and 28.04% in S1HL (Figure 3B‐E). Our results suggest that the SCI model reduces the expression of PNNs, the number of PNNs‐wrapped PV+ cell density in the injured spinal gray matter, and the density of PNNs‐wrapped PV+ neurons in M1 and S1HL, without affecting the PV+ cell density in M1 and S1HL.

Further assessment of PV‐IN activity in the injured spinal cord by in vivo calcium imaging revealed that SCI significantly reduced calcium ion activity in PV‐IN (Figure 3F–L). In addition, we evaluated the changes in spinal cord local PV‐IN activity after the collapse of PNNs structure by the ChABC enzyme, and the calcium imaging results suggested that the enzymatic degradation of PNN structure significantly reduced the calcium activity of PV‐IN (Figure 3N–Q). Therefore, we believe that spinal PNN integrity is involved in regulating PV‐IN activity.

### 3.5 Neurocan maybe an important factor in PNN regulation of PV‐IN in injured spinal cord

**FIGURE 4:**
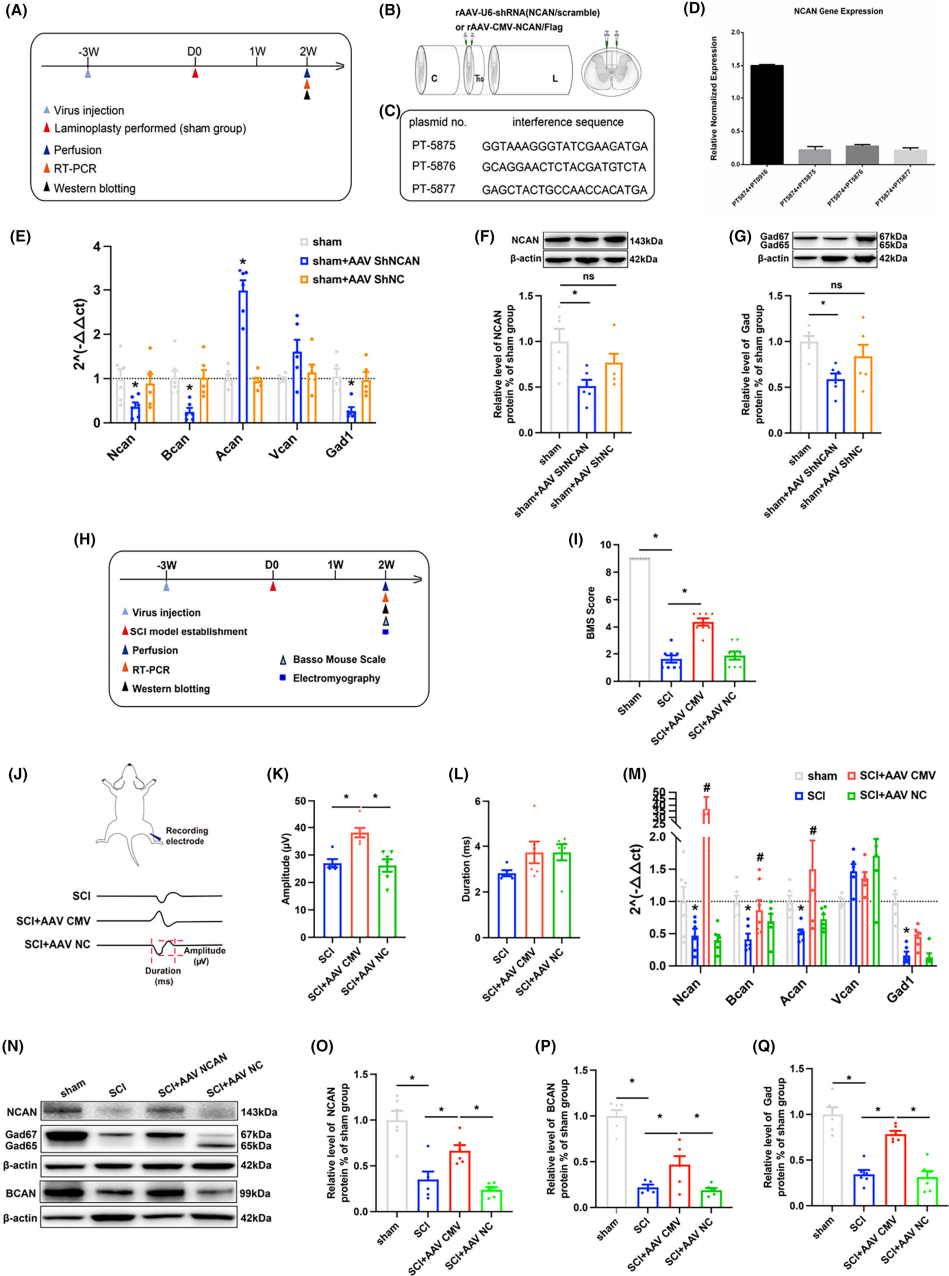
(A, H) Timeline of behavioral detection; (B) Schematic diagram of virus injection; (C, D) NCAN sequence screening sequence and sequence screening efficiency diagram; (E) PCR quantization map of PNN core proteoglycan gene after knocking down NCAN in normal spinal cord tissue (*n* = 6 per group); (F, G) Blot banding and quantification of NCAN and GAD in the spinal cord on Day 14 after NCAN knockdown in the normal spinal cord: The results showed that the expression of NCAN and GAD were down‐regulated after NCAN knockdown in the normal spinal cord (*n* = 6 per group); (I) BMS score of mice in different groups after 2 weeks; (J) The above is the schematic diagram of the EMG, and the below is the representative diagram of the unit EMG of the gastrocnemius muscle in the SCI group, SCI+AAV CMV group and SCI+AAV NC group at Day 14; (K, L) Statistical graphs of amplitude and duration of unit electromyography of gastrocnemius muscle after 2 weeks in different groups of mice; (M) Statistical map of changes in the expression levels of PNN core proteoglycan genes after overexpression of NCAN in injured spinal cord (*n* = 6 per group); (N–Q) Representative strip and statistical map of western blot of NCAN GAD BCAN after overexpression of NCAN in injured spinal cord (*n* = 6 per group). Values are presented as the Mean ± SEM. * indicates *p* < 0.05. ACAN, aggrecan; BCAN: brevican; GAD: glutamic acid decarboxylase; NCAN: neurocan; VCAN: versican.

We further investigated the role of neurocan in SCI by injecting neurocan‐targeted shRNA or overexpressed viruses into the spinal gray matter of adult mice to regulate its expression. Interference virus was injected into the central tubule of the gray matter of the T10 3 weeks before modeling, and perfusion was performed 2 weeks after modeling (Figure 4A,B,H). We also screened the optimal sequence from three gene sequences for shRNA virus packaging (Wuhan BrainVTA) and subsequent experiments (Figure 4C,D). PCR and western blot results showed that the gene and protein expressions of NCAN and Gad were decreased in the spinal cord tissues of mice after targeted injection of NCAN shRNA to knock down the virus, while the expression of control virus was not significantly changed (Figure 4E–G). SCI model was established 3 weeks after injection of NCAN overexpression virus into mouse spinal cord (Figure 4H) to further observe the role of NCAN in injured spinal cord. The results of BMS score showed that the score of BMS in the second week after overexpression of local NCAN in the injured spinal cord was higher than that of model mice (Figure 4I), and the results of electromyography of gastrolnemius muscle showed that the EMG amplitude increased after overexpression of NCAN, and the duration did not change significantly (Figure 4J–L). In addition, PCR and western blot results showed that overexpression of NCAN increased gene and protein expression of NCAN, GAD, and BCAN in local spinal cord tissues (Figure 4M–Q), while no significant changes were observed in control viruses. There was no significant change in the expression level of VCAN gene in the injured spinal cord overexpressed with NCAN, and the expression level of BCAN gene was consistent with that of NCAN. However, local ACAN gene expression was up‐regulated in both the normal spinal cord with NCAN knockdown and the injured spinal cord with NCAN overexpression. In any case, the above results suggest that changes in NCAN can regulate the expression of PNN core protein and play an important role in the structural remodeling of PNN (Figure 4E,M).

**FIGURE 5:**
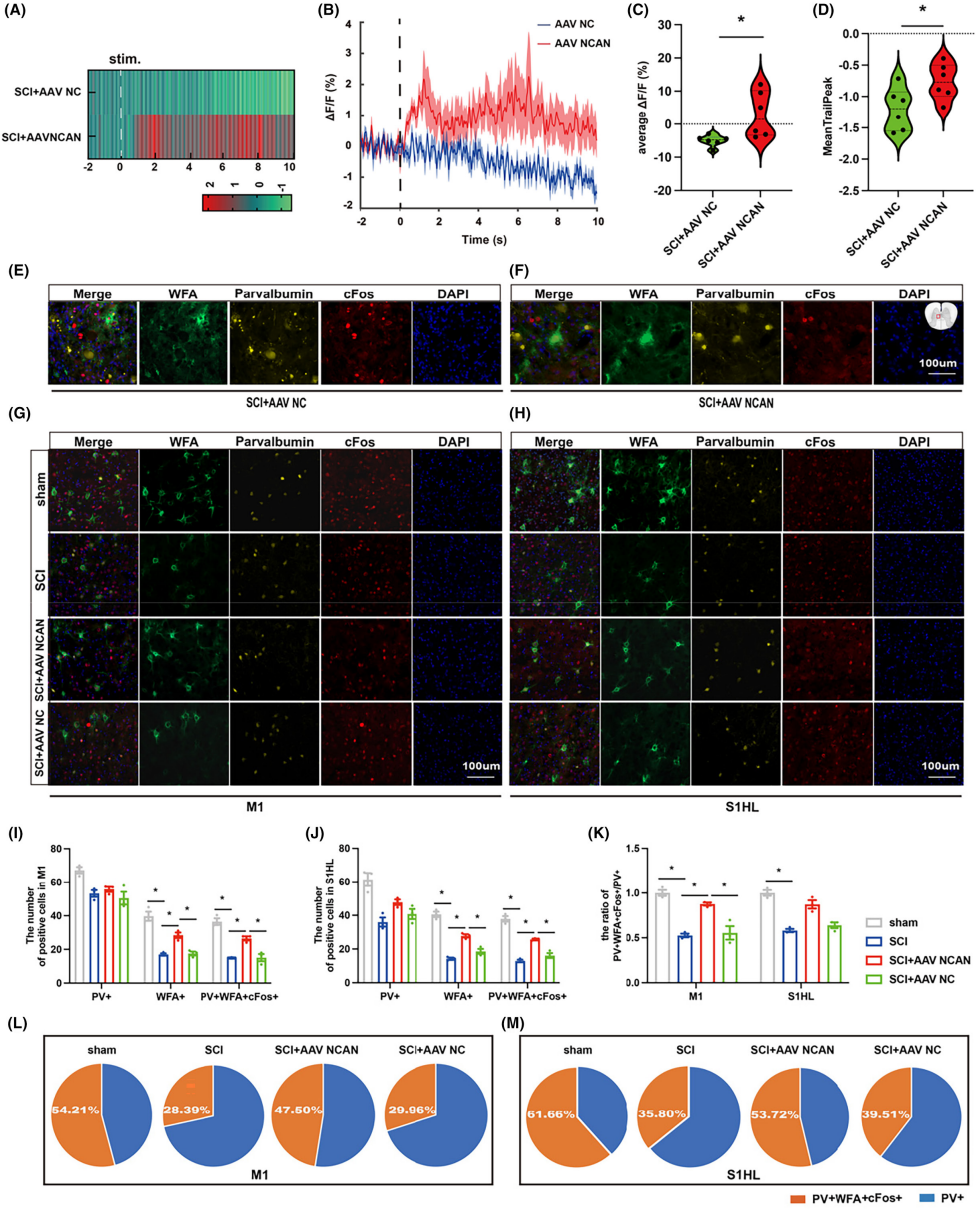
Neurocan is an important factor in PNN's regulation of PV‐IN in injured spinal cord. (A, B) neurocan overexpress virus or control virus intervention on the 14th day of PV‐IN activity changes in the epicenter of damage; (C, D) The average △F/F and meantrailpeak statistics of calcium imaging showed that overexpression of neurocan reduced the decrease in PV‐IN activity; (E, F) Representation of immunofluorescence expression of WFA (green)/parvalbumin (yellow)/cFos (red) in injured areas after intervention of local spinal cord with NCAN‐overexpressed virus or control virus. Nucleus (blue); (G–M) Immunofluorescence expression and quantification of WFA (green), parvalbumin (yellow), cFos (red) and Nucleus (blue) in M1 and S1HL of cerebral cortex in sham group, SCI group, SCI+AAV NCAN group and SCI+AAV NC group (*n* = 3 per group). Values are presented as the Mean ± SEM. * indicates p < 0.05. PV, parvalbumin; WFA, wisteria floribunda agglutinin.

On the contrary, in vivo calcium imaging results suggested that overexpression of local neurocan increase the calcium ion activity of local PV‐IN (Figure 5A–D). Immunofluorescence results showed that overexpression of neurocan could save the low co‐expression of WFA/PV/cFos in the injured spinal cord (Figure 5E,F). In addition, compared with the SCI+AAV NC group, the WFA in the M1 and S1HL regions of the cerebral cortex in the neurocan overexpression group was increased, and the co‐expression rates of WFA/PV and WFA/PV/cFos were up‐regulated (Figure 5G–K). The results showed that the co‐expression of WFA/PV/cFos accounted for 28.39% and 29.96% of M1 and 35.80% and 39.51% of S1HL in NC groups of SCI and SCI+AAV, respectively. It was increased to 47.50% in M1 and 53.72% in S1HL in the SCI+AAV NCAN group. However, PV+ cell density had no significant change (Figure 5L,M). Therefore, we believe that neurocan is an important factor in PNN's regulation of PV neurons and plays a significant role in SCI repair.

### 3.6 Electroacupuncture can benefit for SCI repairment

**FIGURE 6:**
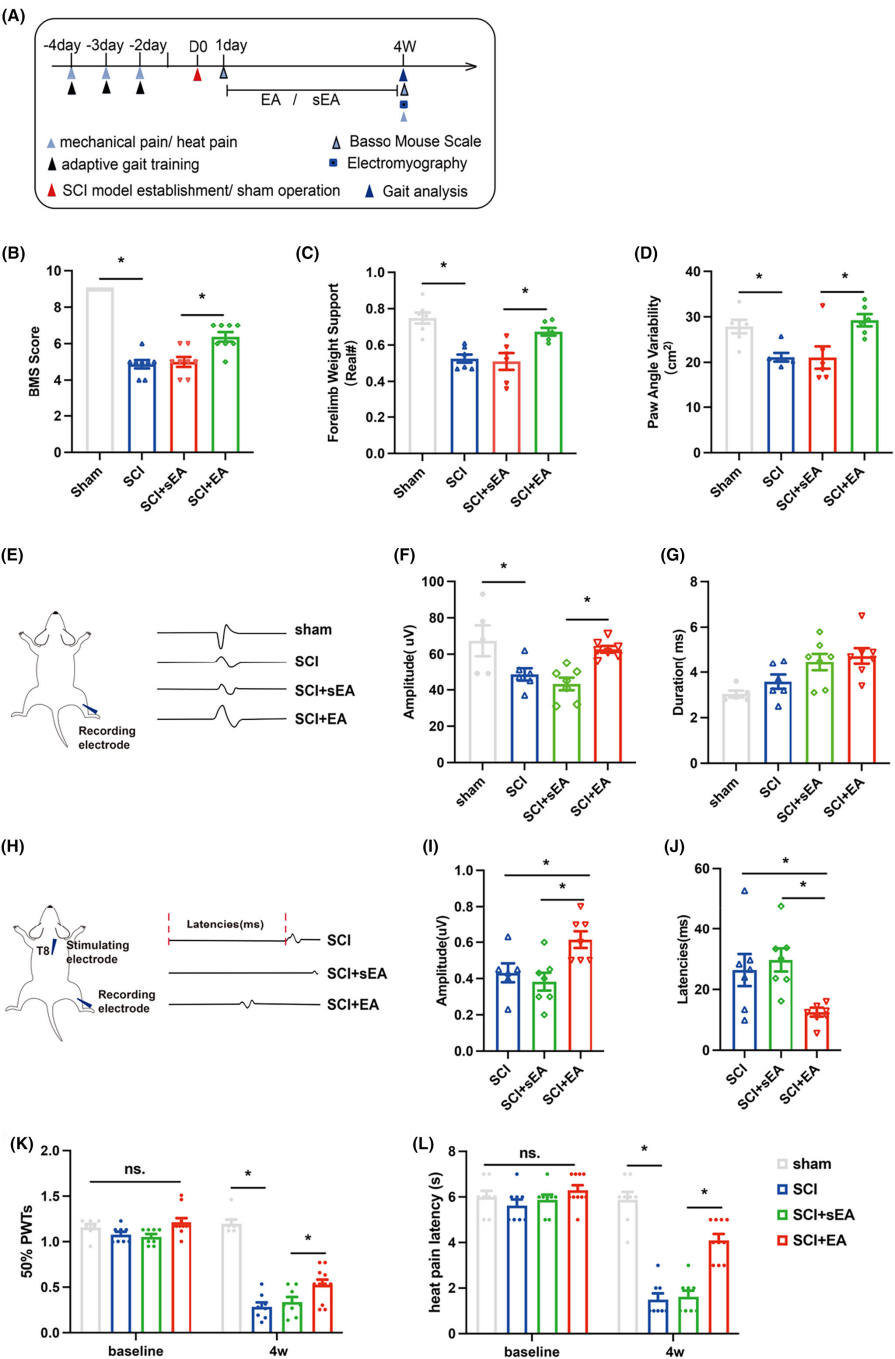
Electroacupuncture promotes functional recovery after SCI. (A) Behavioral detection timeline; (B) Statistical charts of BMS score 4 weeks after operation of mice in different groups; (C, D) Statistical graphs of the forepaw weight support and the paw angle variability in the gait analysis of different groups of mice 4 weeks after surgery; (E) The left is the schematic diagram of the electromyography, and the right is the representative diagram of the unit EMG of the gastrocnemius muscle in the sham group, SCI group, SCI+sEA group, and SCI+EA group at Week 4; (F, G) Statistical plots of unit EMG amplitude and duration of gastrocnemius 4 weeks after operation in different groups of mice; (H) The left is the schematic diagram of EMG, and the right is the representative diagram of spinal cord stimulation‐induced EMG in the SCI group, SCI+sEA group, and SCI+EA group at Week 4; (I, J) The amplitude and latency of gastrocnemius evoked potential at 4 weeks after surgery in different groups of mice were statistically plotted. (K, L) The paw withdrawal mechanical threshold and thermal withdrawal latency of the left hind limb 4 weeks after surgery in different groups of mice. (*n* = 6 ~ 8 per group). Values are presented as the Mean ± SEM. * indicates *p* < 0.05. EA, electroacupuncture; sEA, sham electroacupuncture.

Electroacupuncture has significant neuroprotective effects on the treatment of SCI and its complications. Functional evaluation of SCI mice was performed after four consecutive weeks of electroacupuncture. We found that EA can increase the motor function score of BMS after injury, the forepaw weight support, and the paw angle variability in gait analysis. Moreover, the results of gastrocnemius EMG showed that EA increased the amplitude of unit EMG and spinal cord stimulation evoked potentials, while decreasing the latency of evoked potentials. In addition, EA can improve paw withdrawal mechanical threshold and Thermal withdrawal latency (Figure 6). However, the mechanism of EA therapy to improve nerve function repair after SCI is complex and diverse, and it requires further investigation.

### 3.7 Neurocan may play a key rale to EA in regulating PNN in mice with SCI

**FIGURE 7:**
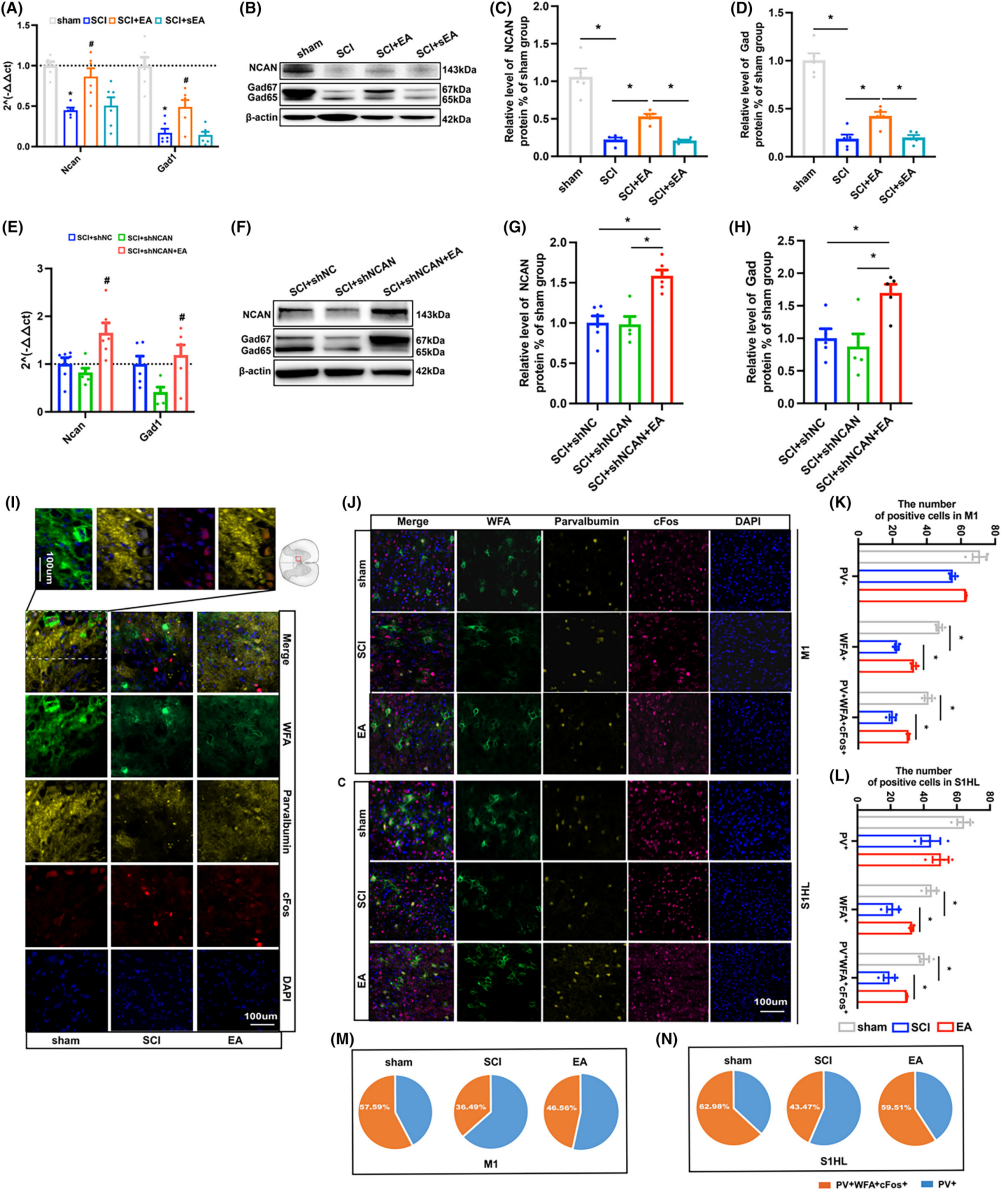
Neurocan may be a key factor in the regulation of PNN by EA in mice with SCI. (A) PCR quantification of NCAN GAD gene expression levels in spinal cord of sham SCI SCI+EA group and SCI+sEA group on Day 14 at the epicenter of injury; (B–D) Western blot bands and quantification of NCAN GAD in the spinal cord of sham group, SCI group, SCI+EA group and SCI+sEA group at the epicenter of injury on Day 14; (E) PCR quantization of NCAN GAD gene expression in SCI+shNC group, SCI+shNCAN group and SCI+shNCAN+EA group at the epicenter of injury on day 14; (F–H) Western blot bands and quantification of NCAN GAD in the spinal cord of SCI+shNC group, SCI+shNCAN group and SCI+shNCAN+EA group at the epicenter of injury on Day 14; (I) Representation of immunofluorescence expression of WFA (green)/parvalbumin (yellow)/cFos (red) around the injured areas in sham SCI and SCI+EA group. Nucleus (blue); (J–N) Immunofluorescence expression and quantification of WFA (green), parvalbumin (yellow), cFos (red), and nucleus (blue) in M1 and S1HL of cerebral cortex in SCI+shNC group, SCI+shNCAN group, and SCI+shNCAN+EA (*n* = 3 per group). Values are presented as the Mean ± SEM. * indicates *p* < 0.05. EA, electroacupuncture; GAD, glutamic acid decarboxylase; NCAN, neurocan; PV, parvalbumin; sEA, sham electroacupuncture; WFA, wisteria floribunda agglutinin.

Gene and protein levels of NCAN and Gad in the injured spinal cord were observed. It was found that EA promoted the expression of NCAN and Gad in the injured spinal cord (Figure 7A–D). Immunofluorescence also suggested that EA could increase the co‐expression of WFA/PV in the spinal cord (Figure 7I), the density of WFA and the colabeling rate of WFA/PV/cFos in cerebral cortex M1 and S1HL (The colabeling rate of WFA/PV/cFos in sham group at M1 accounted for 57.59% of the total PV+ cells. In the SCI group, it was reduced to 36.49% and increased to 46.56% after EA therapy. The co‐standard rate of WFA/PV/cFos in the sham group of S1HL was 62.98%, which decreased to 43.47% in the SCI group and increased to 59.51% after EA treatment), instead of affecting PV+ cell density (Figure 7J–N). We further performed EA on SCI model mice with neurocan knockdown was found to reverse the downward trend of NCAN and Gad (Figure 7E–H).

## 4 DISCUSSION

Studies in animals with SCI have shown significantly reduced PNN density around lumbar motor neurons,^34^ rearrangement of PNN structures,^35^ atrophy of inhibitory GABA interneurons, and down‐regulation of parvalbumin cell expression.^36^ These are not only detrimental to functional recovery after injury but also severely affect neuronal activity in the injured area. However, physical rehabilitation exercises can reverse this effect of PNN reduction and remodeling.^34^ Symptoms of neurological dysfunction caused by atrophy and down‐regulation of interneurons can be alleviated by the targeting of inhibitory interneurons at the injury site,^37^, ^38^, ^39^ which effectively promotes the recovery of motor function after SCI.^40^ This suggests that PNN and inhibitory interneurons play an important role in spinal cord injury and its repair.

Previous studies have shown that PNN is vital in GABAergic synaptic plasticity,^41^, ^42^ the disruption of the PNN structure can lead to decreased excitability of PV‐IN.^43^, ^44^, ^45^ At the network level, depletion of PNN affects gamma oscillations (30–80 Hz) and sharp wave ripples (SWRs), since PV neuron activity is critical for the generation of gamma oscillations and SWRS.^46^, ^47^ Tightly structured PNN form a high density of negative charge around PV‐IN, which can not only attract high concentrations of Na, K, or Ca ions to help PV‐IN activity during the rapid peak activity,^48^ but also protect PV‐IN from the damage of extracellular stressors.^49^ Conversely, the destruction of the PNN structure makes PV‐IN more susceptible to the influence of external stressors.^50^, ^51^ These findings support the scientific claim that decreased neuronal activity of local PV‐INs in the injured spinal cord is strongly linked to the destruction of peripheral PNN. Consistent with other previous findings, our study found that the genes encoding parvalbumin and inhibitory interneuronal receptors in both normal and injured spinal cords were affected after ChABC intervention, and inhibitory interneurons were the crucial targets of PNN in SCI mice by RNA‐Seq analysis. Furthermore, the Ca^2+^ activity of local PV‐IN was significantly weakened in the normal spinal cord after the ChABC intervention. At the same time, similar changes were observed in the injured spinal cord with the destruction of PNN, indicating that the neuronal activity of PV‐IN in the spinal cord depends on the integrity of PNN.

However, PNN is a complex network structure, and the key factors of its function must be further studied. Surprisingly, this study found that the gene encoding the core proteoglycans showed the same trend regardless of whether the spinal cord was treated with ChABC in normal or injured, except that NCAN showed the opposite trend. Studies have shown that neurocan is diffusely dispersed in the central nervous system and play an important role in the which production of PNN,^52^ closely associated with the pathological mechanisms of brain injury, memory, depression, and other psychiatric disorders.^53^, ^54^, ^55^, ^56^ Hence, we performed PPI analysis and found that NCAN indeed regulated inhibitory interneurons function via parvalbumin. This suggests that NCAN in PNN is important for the repair ability of SCI. To elucidate the role of NCAN in regulating parvalbumin in the spinal cord, we treated the normal spinal cord with NCAN knockdown virus and overexpressed NCAN in the injured spinal cord. The corresponding changes of other PNN proteoglycans, parvalbumin, and GAD were observed. Notably, overexpression of lesion‐localized NCAN improved motor function recovery after SCI and enhanced local PV‐IN activity. We also observed PNN‐specific WFA staining^57^ in the cerebral cortex and found that the WFA/parvalbumin/cFos co‐labeling rate decreased after the model, increasing as local NCAN overexpression improved motor function. Indeed, Parvalbumin expression measured by immunofluorescence is widely considered to reflect neuronal activity in PV cells.^58^ For example, the cumulative frequency of PV fluorescence in single cells suggests that the mean PV fluorescence of PNN+/PV+ neurons is significantly higher than that of PNN‐/PV+ neurons.^59^ However, this is not always consistent with the electrophysiological readings. In addition, studies have confirmed that NCAN can regulate mRNA and protein expression of PNN‐related molecules and GAD65/67 expression and synaptic transmission in GABAergic synapses.^52^ The specific knockout of PNNs core proteins, especially NCAN and BCAN, can lead to decreased structural stability and number of PNN and significant loss of PV‐IN wrapped in PNN, reducing the number of inhibitory interneuron synapses and weakening PV‐IN activity.^60^, ^61^ This could also be due to NCAN influencing neuronal development and apoptosis in conjunction with specific receptors on neuronal cell membranes and cell adhesion molecules.^62^, ^63^ In conclusion, we believe that NCAN regulates the neuronal activity of PV‐IN and is an important factor in maintaining the functional remodeling of PNN in mice with spinal cord injury.

According to traditional Chinese medicine qi theory, paraplegia caused by SCI belongs to the “impotence syndrome” category. It is caused by damage to the Governor meridian, blockage of meridians, and muscle loss in support of qi and blood. The Jiaji point (EX‐B2) is located between the governor meridian and the bladder meridian. EX‐B2 electroacupuncture can dreg the governor meridian and the bladder meridian at the same time, exciting Yang qi so that it can reach the extremities to improve the local neuronal microenvironment of the injured spinal cord and promote long‐term neurological function recovery in SCI patients.^64^ In the present study, EA reversed the loss of neurocan in PNN after injury, enhanced, and elevated the activity of parvalbumin coated with PNN, and improved motor and sensory dysfunction after SCI. Based on these findings, we propose that EA can effectively improve the structural and functional destruction of PNN after injury through neurocan to balance the neuronal microenvironment, thereby promoting repair after SCI.

The response of PNN to external stimuli is generally observed, but whether the reduction of PNN early after SCI is functionally relevant to the formation of glial scars later in life remains to be seen. On the other hand, the role of PNN in the central nervous system is not only limited to inhibitory interneurons but also related to glutamatergic and monoaminergic neurons. The spinal cord neural circuits are complex and interconnected. Understanding the complex mechanisms of PNN component generation and breakdown, as well as linking these processes to physiological changes at the molecular and network levels, is critical for further understanding the implications of PNN remodeling associated with neurological impairment and aiding in the development of new therapeutic approaches for SCI. As a safe and low‐risk treatment, EA is expected to become a new treatment for SCI.

## 5 CONCLUSION

Our study demonstrates that neurocan in the core proteoglycans of PNN can regulate the neuronal activity of PV‐IN and is vital in maintaining the functional remodeling of PNN in mice with SCI. In addition, EA can salvage the loss of stable structure and functional destruction of PNN after SCI by regulating neurocan expression in PNN, promoting motor and sensory function recovery.

## AUTHOR CONTRIBUTIONS

Rong Hu and Kelin He contributed equally to this work as co‐first authors. Ruijie Ma, Rong Hu, and Kelin He designed this study. Rong Hu, Xingying Wu, Mengting Shi, Bowen Chen, and Jieqi Zhang completed this study. Rong Hu, Mengting Shi, and Yi Chen analyzed the data. Rong Hu, Kelin He, and Xingying Wu wrote the manuscript. Yi Chen, Bowen Chen, and Lei Wu participated in the manuscript revision. Ruijie Ma validated the manuscript. All authors had read and approved the final version of the paper.

## CONFLICT OF INTEREST STATEMENT

The authors declare that there is no conflict of interest regarding the publication of this paper.

## Supporting information


Data S1.

Figure S1.
Figure S2.

## Data Availability

All the data used to support the findings of this study are included within the article. The RNA‐seq datasets generated and/or used during the current study have been deposited and are available from National Center for Biotechnology Information (NCBI) repository with the accession code PRJNA941011. It will be accessible with the following link after the indicated release date: https://www.ncbi.nlm.nih.gov/sra/PRJNA941011
