# Supplementary material for: Electroacupuncture promotes the repair of the damaged spinal cord in mice by mediating neurocan‐perineuronal net
Source: CNS Neurosci Ther. 2023 Nov 10;30(1):e14468. doi: 10.1111/cns.14468 (PMC10805400; doi:10.1111/cns.14468)

Full unedited blot for Figure 1

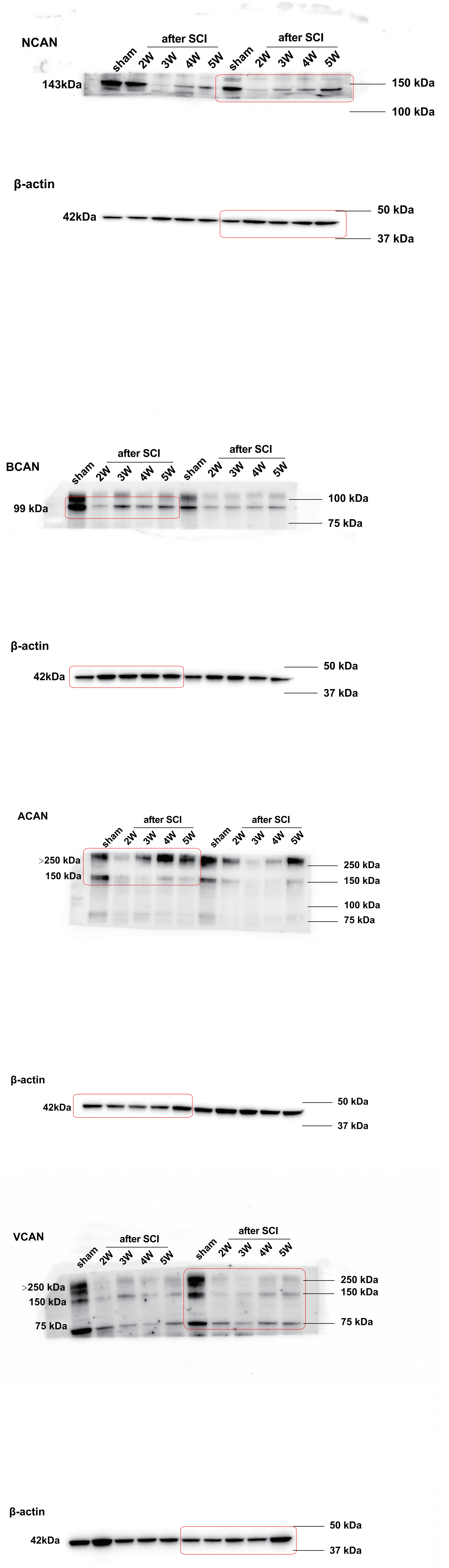

Full unedited blot for Figure 2

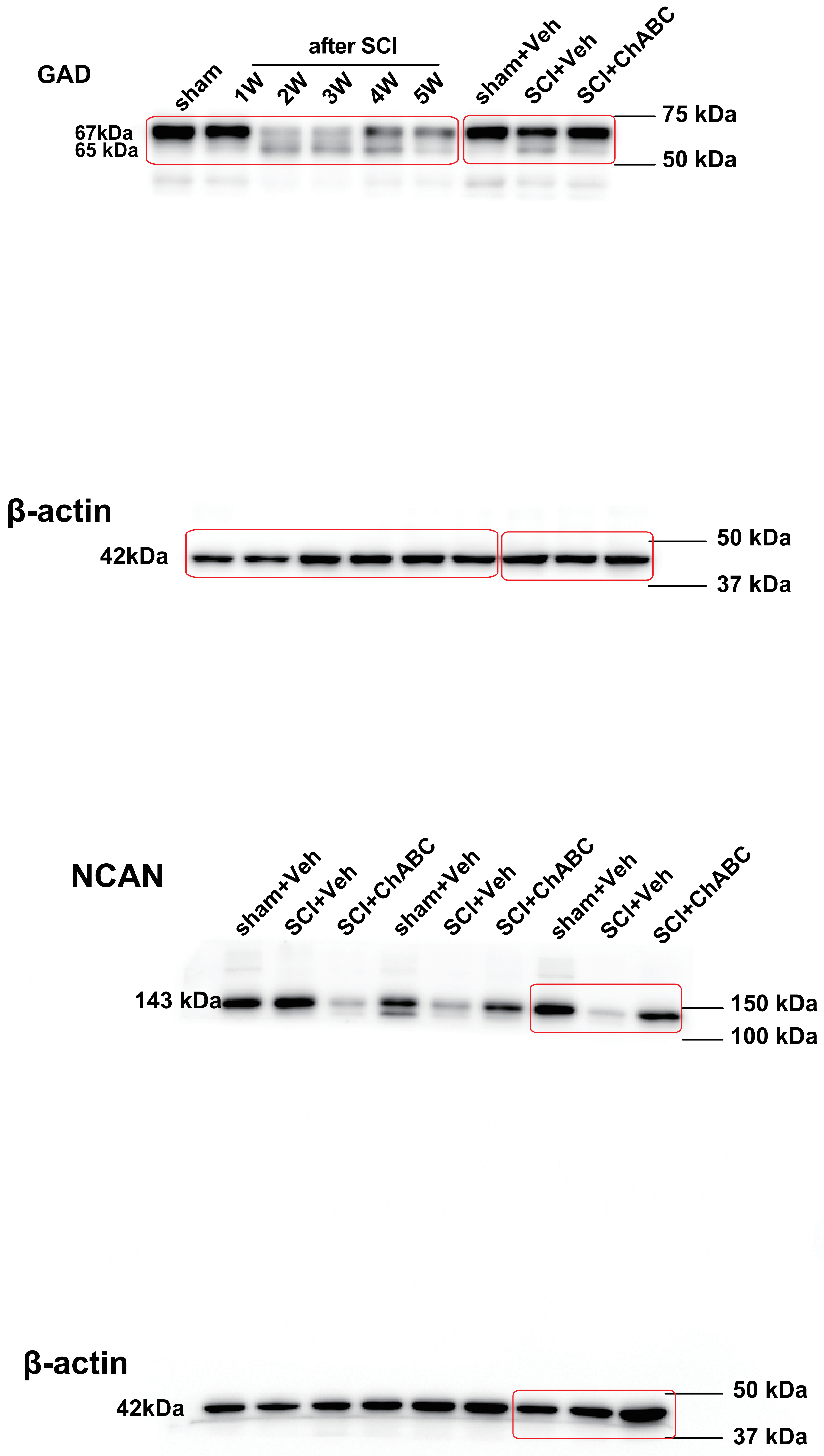

Full unedited blot for Figure 4

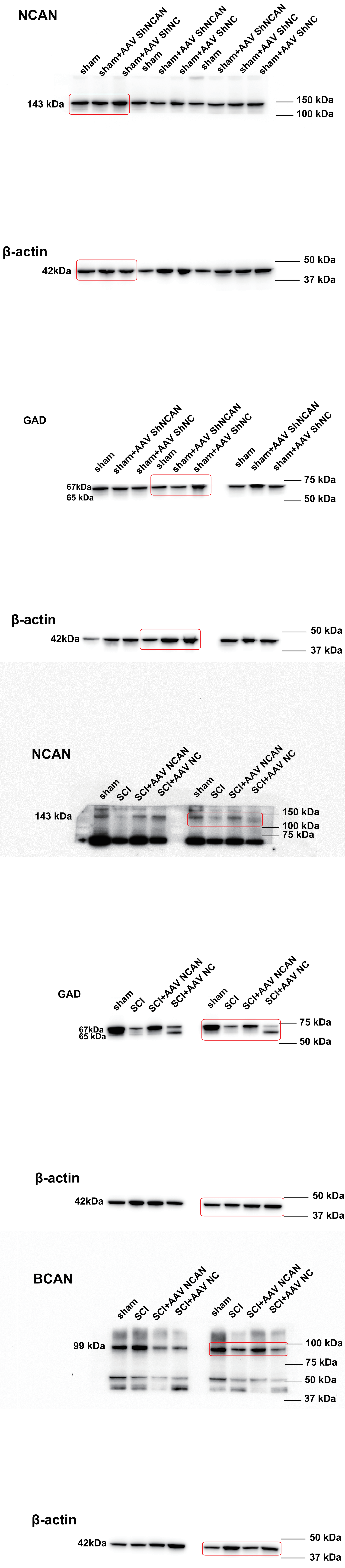

Full unedited blot for Figure 7

NCAN

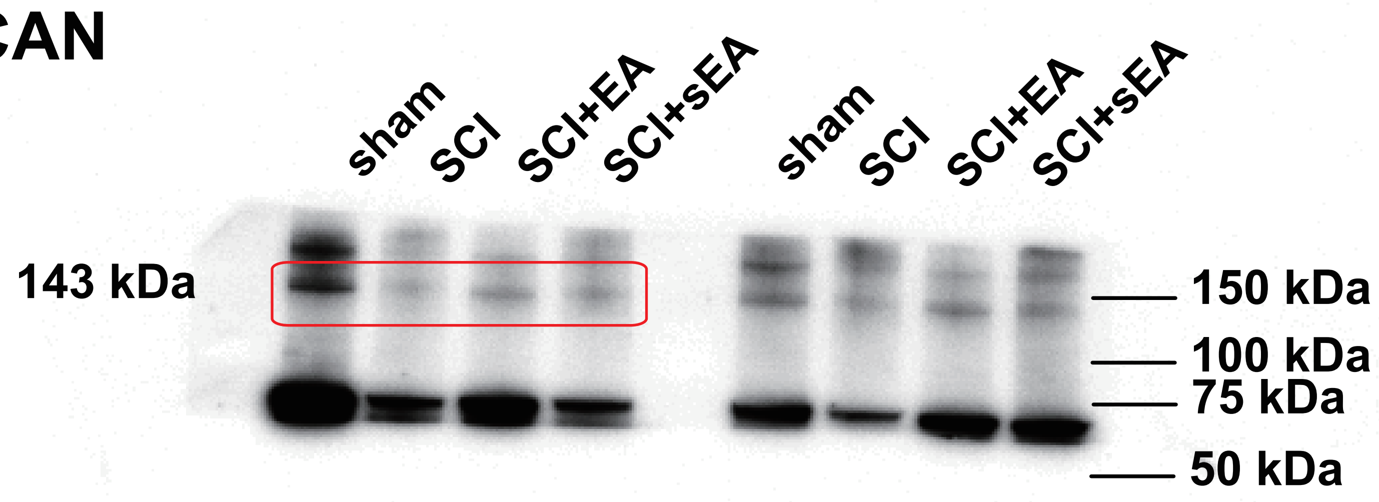

GAD

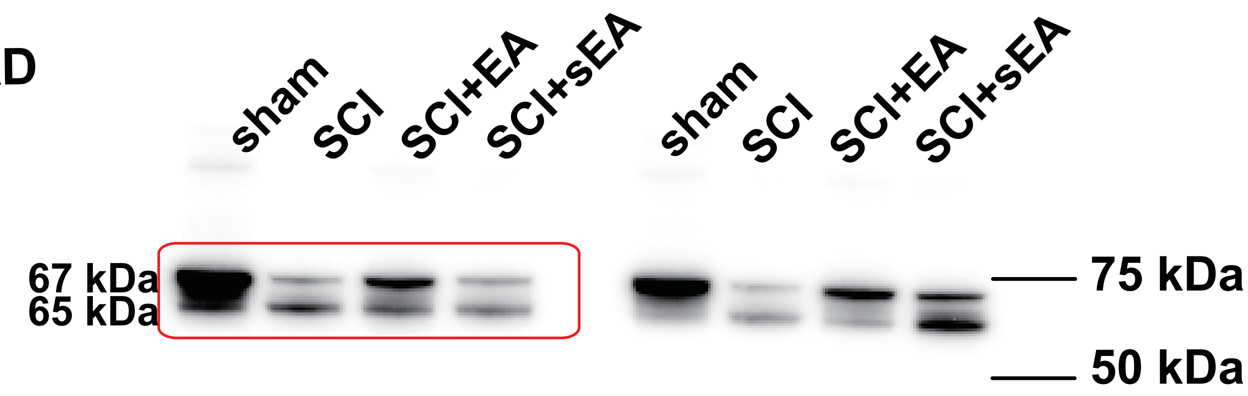

β-actin

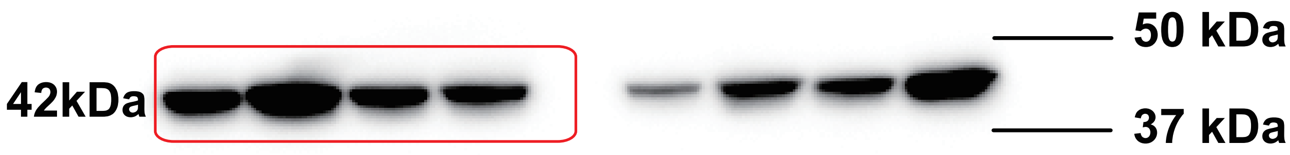

NCAN

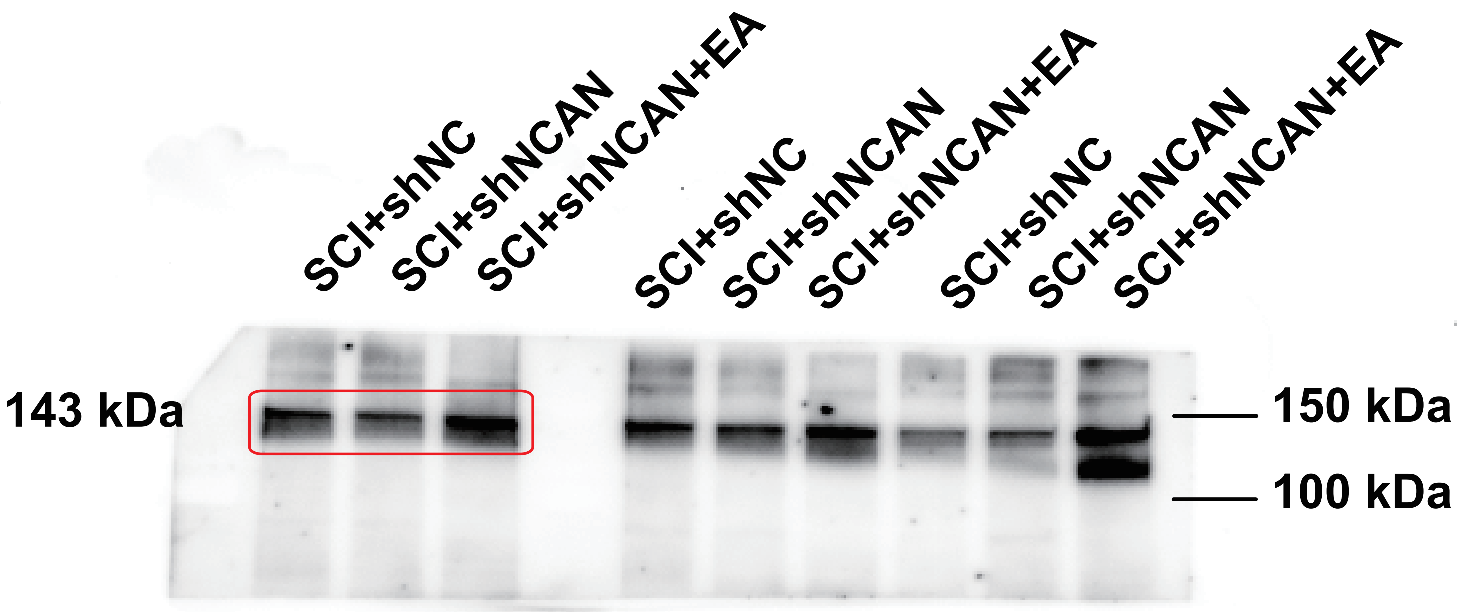

GAD

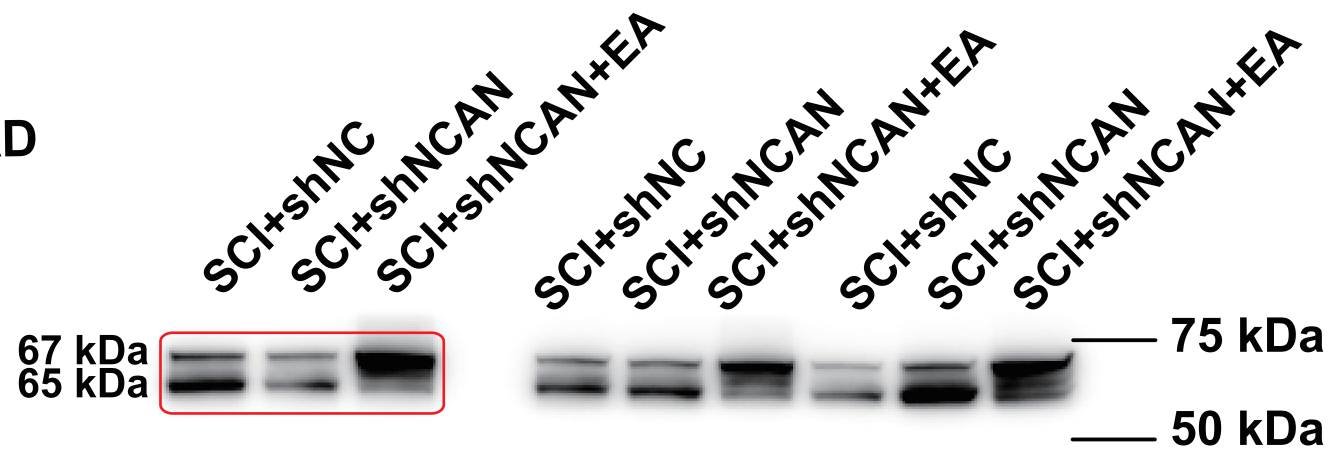

β-actin

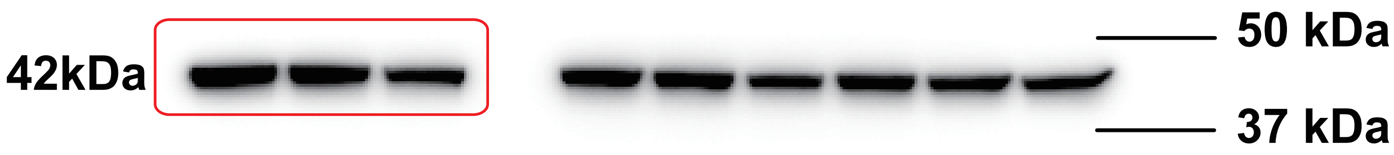

Supplement: Supplementary file 1 — Data S1. [file CNS-30-e14468-s001.zip › Supplementary documents.pdf]
