# Supplementary material for: Electroacupuncture promotes the repair of the damaged spinal cord in mice by mediating neurocan‐perineuronal net
Source: CNS Neurosci Ther. 2023 Nov 10;30(1):e14468. doi: 10.1111/cns.14468 (PMC10805400; doi:10.1111/cns.14468)
Supplement: Supplementary file 2 — Figure S1. Figure S2. [file CNS-30-e14468-s002.docx]

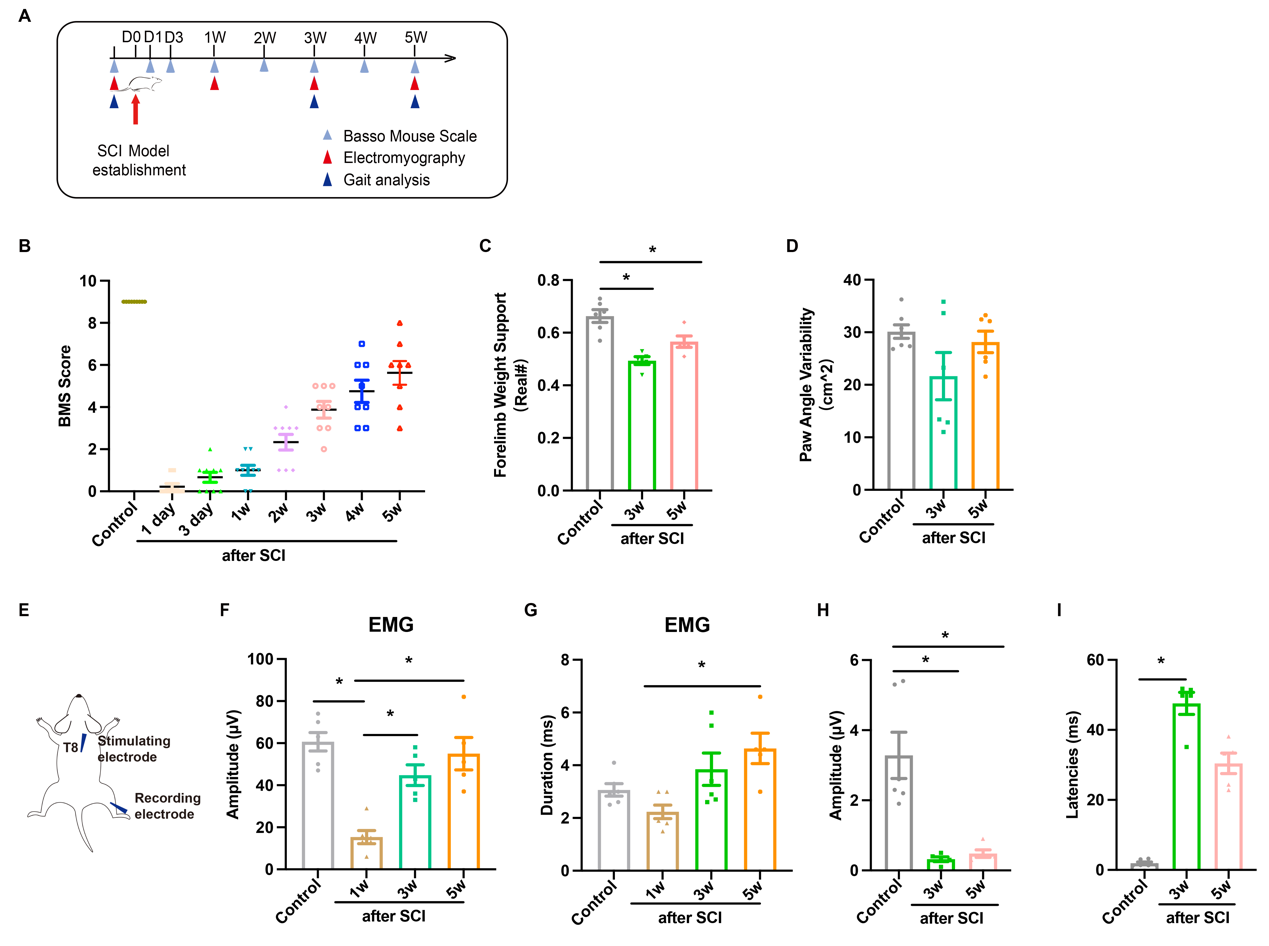


Supplementary Figure1. Motor dysfunction of lower limbs in mice after successful establishment of spinal cord injury model. A: Experimental timeline; B: BMS motor function scores at different time points after spinal cord injury; C D: Statistical graphs of the forepaw weight support and the paw angle variability in gait analysis at different time points after injury; E: Electromyogram diagram; F G: EGM amplitude changes at different time points; H I: Quantification of evoked potential latency and amplitude change.(n = 6 ~ 8 per group). Values are presented as the Mean ± SEM. * indicates p < 0.05.


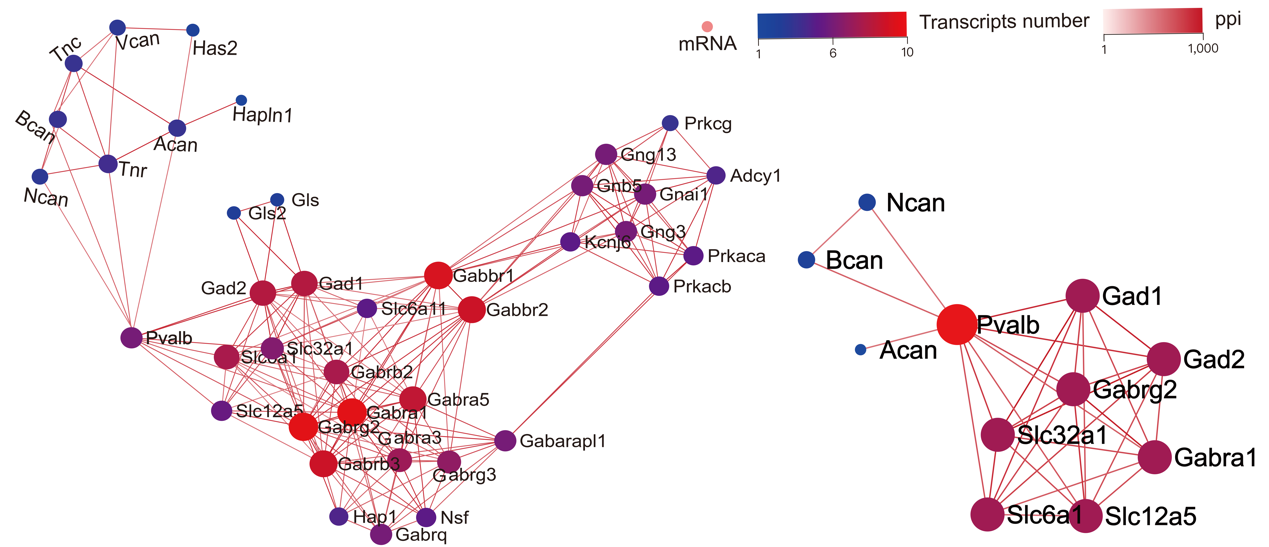


Supplementary Figure2. PPI network map of PNN structural protein and GABAergic synapse enrichment gene.
